# Valerianate of Quinia

**Published:** 1845-10

**Authors:** 


*Valerianate of Quinia.*—Dr. Devay, in an essay on the Valerianate of Quinia, published in the Gazette Medicale, recommends it as equal to the sulphate in its anti-periodic eflects, and much superior in its nervo-sthenic properties.

*Preparation.* To a concentrated alcoholic solution of quinia, add a slight excess of valerianic acid. Dilute the solution with twice its volume of distilled water, and evaporate in a stove, with a temperature not above 120° Faht. During the evaporation, handsome crystals of the valerianate are formed, arranged singly or in groups. Dry the salt in the open air.

The valerianate of quinia may be also obtained by decomposing sulphate of quinine, with the valerianate of lime or baryta, both being in solution in dilute alcohol.

*Chemical and Physical Characters.* The Prince of Canino, who was the first to prepare this salt, ascertained it to be composed of valerianic acid, 1 equiv.; quinia, 1 equiv., and water, 2 equiv., of which one constitutes its water of crystallization. The crystalline form seems to be variable, sometimes hexahedral or octohedral, and at others forming rhomboidal tables, or agglomerated in light, silkey masses.

The odor slightly resembles that of valerianic acid; and the taste bitter. It dissolves readily in water, but alcohol and olive oil are better solvents. It is decomposed by the mineral, and most of the organic acids. It loses one equiv. of water when heated at 90°, and then melts like a resin.

*Dose, and Mode of Administration.*—Being readily decomposed, it is recommended to administer it in a simple mixture, as with gum mucilage. Three and a half ounces of the mucilage of gum arabic, will dissolve 8 grains of the salt. A grain may be given at a dose.

One of the great advantages of this salt is supposed to be its ready solubility in olive oil, in which form it may be applied endermically over the region of the spleen. The form recommended is:

℞ Olive oil, ℥ ii;
Valerianate of Quinine, grs. xvi.—dissolve.

M. Devay reports many cases; some of which were severe and complicated intermittents, which the sulphate had failed to relieve, and which yielded when the valerianate was used. From 1½ to 6 grains, constituted the per diem quantity in the various cases.

From the experiments made, M. Devay concludes, 1st. Valerianate of quinine is superior as an anti-periodic to the sulphate, in consequence of its nervo-sthenic properties, and because it acts in smaller doses.

2d. Given alone, it is equivalent to cinchona combined with the *nevritiques.*

3d. In the worst fevers (the malignant ataxic) it is thought to act most beneficially, by its specific properties.—*New Orleans Medical and Surgical Journal.*

